# A Case of Temporomandibular Joint Ankylosis With Adenoid Hypertrophy and Associated Airway Challenges

**DOI:** 10.7759/cureus.58153

**Published:** 2024-04-12

**Authors:** Samarpan Patel, Sanjot Ninave

**Affiliations:** 1 Anaesthesiology, Jawaharlal Nehru Medical College, Datta Meghe Institute of Higher Education and Research, Wardha, IND

**Keywords:** nasal fiber optic intubation, difficult airway management, ankylosis, temporomandibular joint ankylosis, pediatric anaesthesia

## Abstract

Temporomandibular joint (TMJ) ankylosis is a form of TMJ condition that causes mouth opening limitation, ranging from partial reduction to total immobilization of the jaw. Bony and fibrous ankylosis is most commonly caused by trauma, although it can also happen as a result of surgery, local or systemic infections, or systemic diseases. Childhood TMJ produces facial deformities, which increase with growth and have a major detrimental impact on the patient's psychological development. Each patient with TMJ ankylosis must have a history, physical examination, and radiographic examination in order to determine a definitive diagnosis, severity, involvement of surrounding tissues, and, ultimately, treatment planning. Technical challenges and a high recurrence rate make treating TMJ ankylosis challenging. Intubating a young child with TMJ ankylosis is a difficult job, which is exacerbated by limited mouth opening. This case report describes a five-year-old boy who reported an inability to open his mouth, diagnosed as TMJ ankylosis, and managed in the absence of an appropriately sized tracheostomy tube.

## Introduction

The temporomandibular joint (TMJ) is a complicated skeletal structure that is essential for optimal jaw function. It is also regarded as the body's most active functional joint. Fundamentally, there is bilateral (BL) synovial articulation between two joints that connect below the mandibular to the temporal bone of the head. Despite their BL articulation, these joints function as a single unit and are therefore dependent on one another. The TMJ is regarded as a unique form of joint in the body [1]. Ankylosis is a hardening of a joint, which causes rigidity and aberrant adhesion in the bones of the joint, that is minimal movement of the condyle, which may hamper the ability to open the mouth partially or completely. Most instances are caused by the fusion (bony or fibrous) of the mandibular condyle to the base of the skull [2]. Trauma is one of the leading causes of bony and fibrous ankylosis.

The leading imaging technique is computed tomography [3]. The connection of ankylotic mass to the anteroposterior and mediolateral dimensions and the glenoid and middle cranial fossa may be examined accurately and clearly. As a result, it provides a very precise and descriptive representation in all planes [4,5]. Ankylosis is most commonly treated with surgery. No single surgical method has been proven to be completely successful. The literature describes a variety of operations, such as condylotomy, simple as well as interposition arthroplasty, utilizing deep temporal fascia, ear cartilage, fascia lata, alloplastic material, and joint reconstruction using costochondral graft (CCG), fibula, iliac, clavicle crest, and metatarsal head [6,7].

## Case presentation

**Figure 1:**
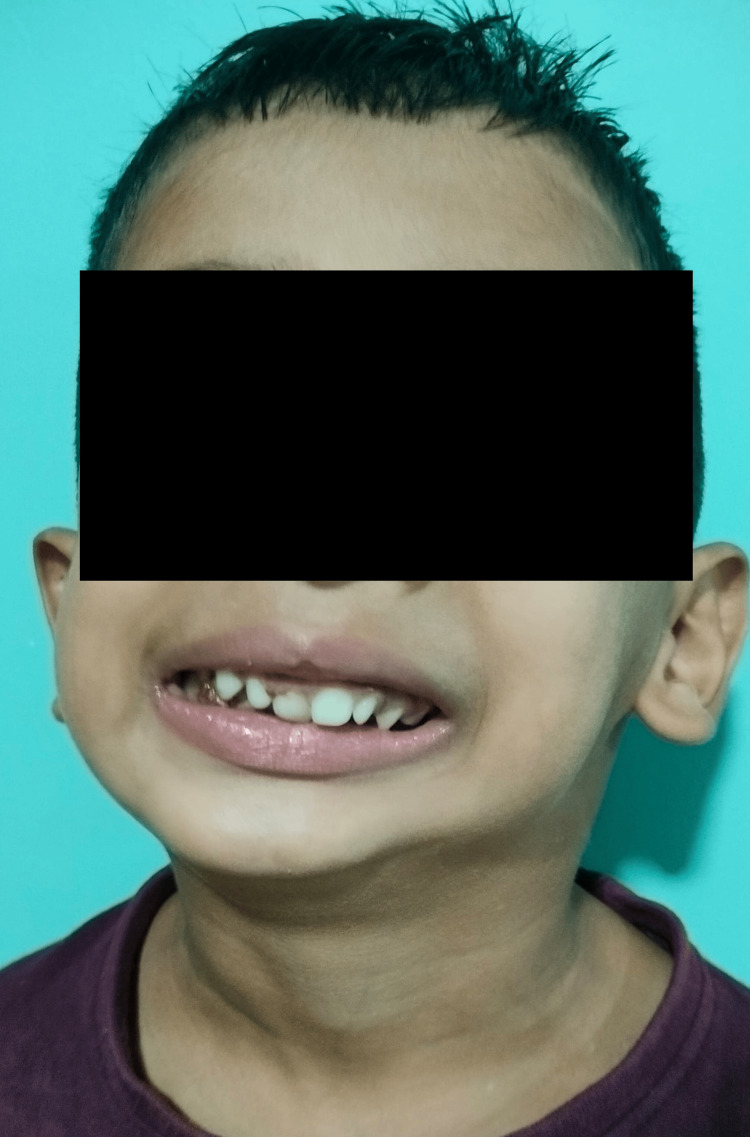
General features of child.

A five-year-old male child of 15 kg, as shown in Figure 1, presented with complaints of progressive reduction in mouth opening over the past four years; mouth breathing for two years, which was gradually progressive and was associated with snoring during nighttime, aggravated by cough and cold; and difficulty in speech for the last two years. There was no history of trauma. The parents reported how the mouth opening had shrunk with time. When this constraint interferes with feeding and influences the child's health, seeking treatment for the issue becomes necessary.

The patient was born after completing a gestational period of nine months by vagino-vertex delivery. All milestones of development were achieved on time. The patient had a known case of hypothyroidism for one year and was on tablet levothyroxine 25 mg. There is no history of hypertension, diabetes mellitus, asthma, or jaundice.

Upon general examination, pulse was 82/min, respiratory rate 18/min, blood pressure (BP) 112/72 mmHg. Systemic examination revealed first and second heart sounds. Bilateral air entry was equal, abdomen soft and non-tender, and the patient was conscious.

**Figure 2:**
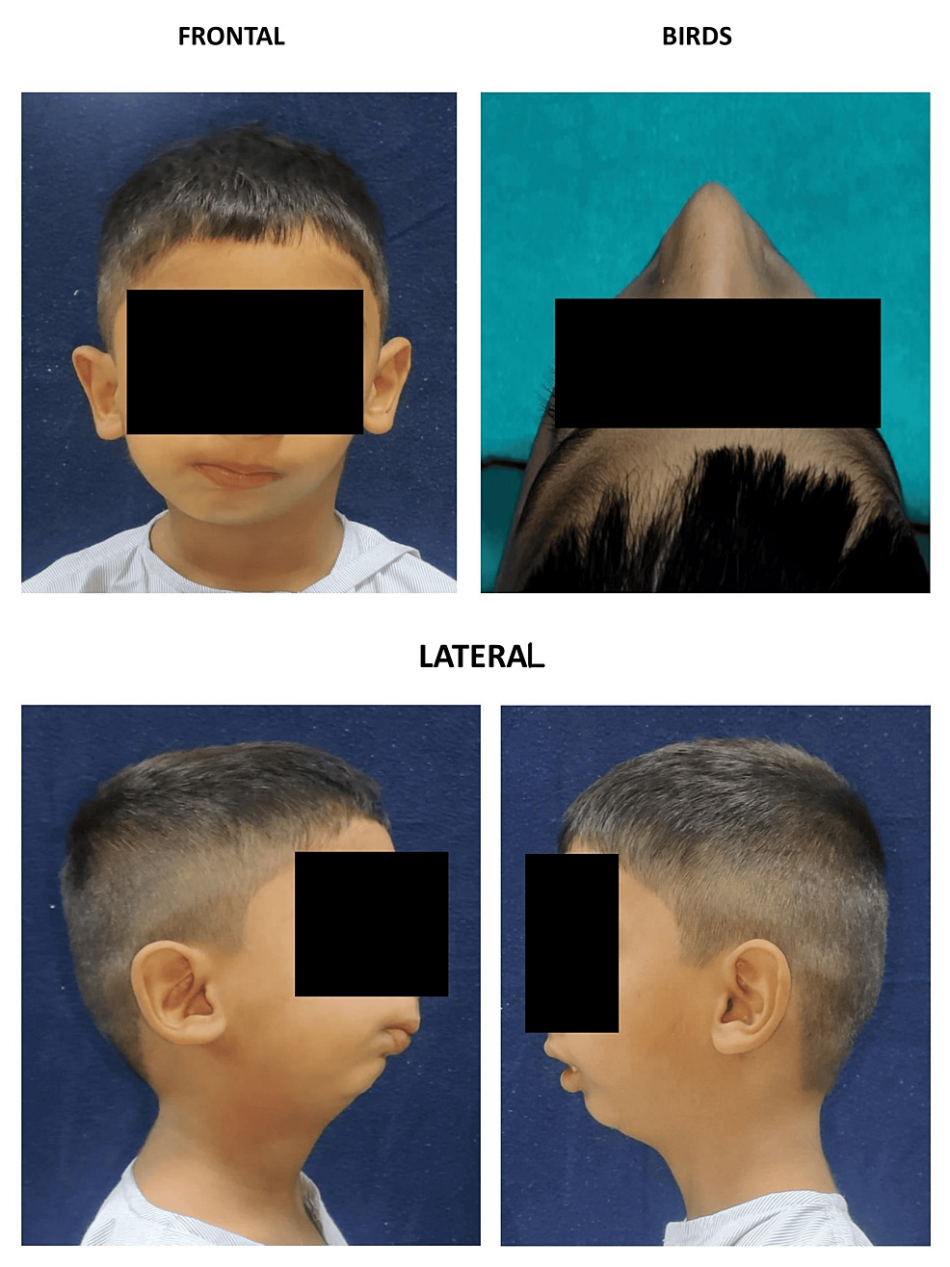
Facial dysmorphism.

On airway examination, the patient had no mouth opening, minimal neck extension was given, and the nasal patent was not checked since there is a risk of epistaxis that can complicate fiber optic vision; therefore, direct vision and an expert hand were used for safe and clear fiberoptic (FO) intubation. For patency, an ENT opinion was taken. The patient's face was grossly asymmetrical due to deviation of the chin towards the right side, flatness over the left side, and fullness over the right side. The mandible was retrognathic, and a convex facial profile is shown in Figure 2. Examination of the TMJ revealed no mouth opening and restricted lateral excursive jaw movements. Lips appeared potentially competent, but there was the loss of the mentolabial sulcus.

**Figure 3:**
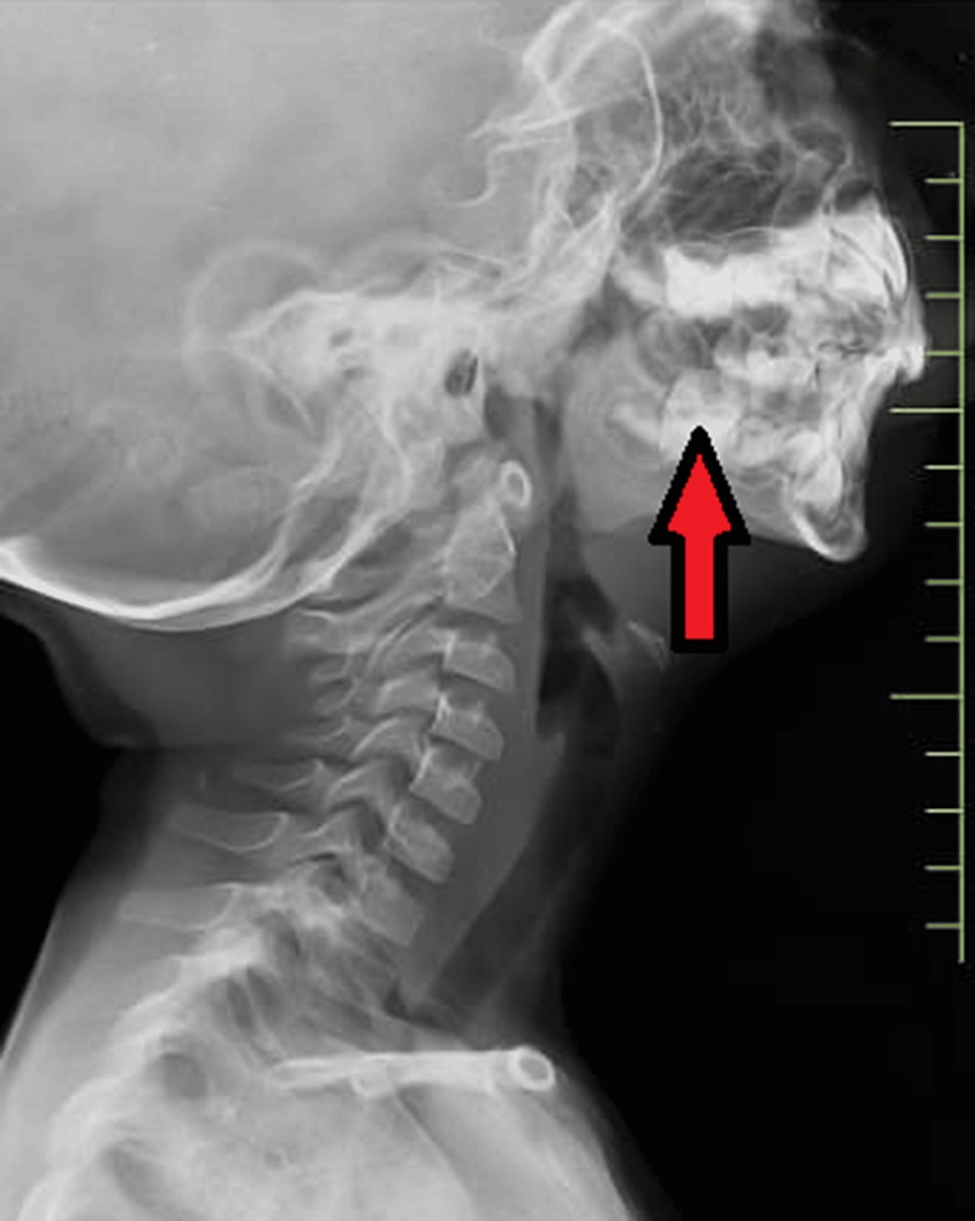
X-ray of the nasopharynx showing grade 3 adenoid hypertrophy.

Lab investigation was within normal limits. The chest X-ray and ECG were within normal limits. X-ray nasopharynx lateral view was done and revealed grade 3 adenoid hypertrophy (Figure 3).

**Figure 4:**
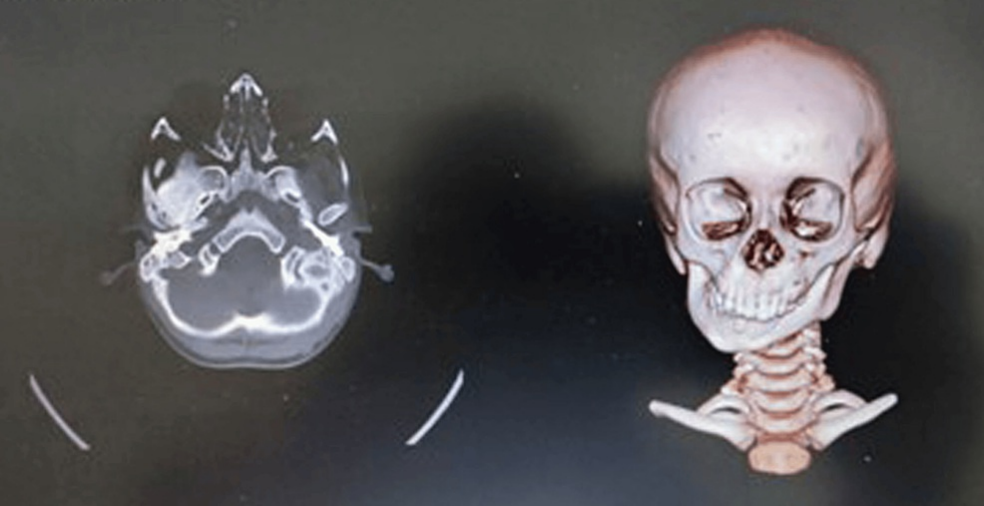
CT and 3D reconstruction of head confirming TMJ ankylosis. TMJ: temporomandibular joint.

A restricted TMJ space and signs of right TMJ ankylosis were discovered, later confirmed by CT scans and third image reconstruction of the head, which showed restricted growth on the right side, condyle, and glenoid fossa fused, as shown in Figure 4.

The patient was scheduled for surgery after obtaining high-risk consent from the parents. Due to the difficulty in arranging the tracheostomy tubes of sizes 4.5, 5, and 5.5 mm, the procedure was delayed many times as the patient had no mouth opening and adenoid hypertrophy. It was planned to perform left nasal intubation under conscious sedation through a pediatric fiberoptic bronchoscopy (FOB). Our plan B was to proceed with tracheostomy and if fails then for rescue ventilation emergency cricothyroidotomy. ENT opinion was done for a difficult airway, and ENT surgeons were kept on standby if a tracheostomy or emergency cricothyroidotomy was required.

Fasting guidelines were followed as per the 6-4-2 rule, which is six hours for solid food, four hours for breast milk, and two hours for clear fluids. The pediatric resident secured an IV line. Pre-operative room injections (inj) of glycopyrrolate (0.004 mg/kg), midazolam (0.05 mg/kg), and ketamine 10 mg were given. The patient was shifted to the operating room. Monitors were attached, vitals were noted, and ASA standards of monitoring were followed. Then, the patient was pre-oxygenated with 100% FIO_2_ for three minutes with a Jackson-Rees (JR) circuit. Pre-induction was performed with inj fentanyl (1-2 mcg/kg). The patient was kept under conscious sedation with a ketamine dose of 2 mg/kg. A total of 20 mg was given, 10 mg for pre-induction, then 5 mg twice for maintenance of conscious sedation. Then, the patient was nasally intubated with a 5 mm cuffed flexometalic tube with the help of a pediatric FOB of size 3.8 mm.

Intubation was difficult because the patient had a large epiglottis and the cord was anteriorly placed. Also, the curvature of the pediatric FO made it difficult to negotiate both epiglottis and anteriorly placed cords. Also, the vision was getting poorer with time. Supplemental oxygen was given in the right nostril with nasal prongs while the patient was undergoing FO intubation under sedation, and an airway block or local anesthetic spray was given. The duration of FO intubation was 1 minute 30 seconds. With patience and an expert hand, it was successful in the end. 

Once the tube was confirmed with five-point chest auscultation and capnography, inj propofol (2 mg/kg) and inj atracurium (0.5 mg/kg) were given. The patient was maintained on O_2_, N_2_O, and sevoflurane. The patient was given fentanyl with inj paracetamol for intraoperative analgesia and inj paracetamol post-operatively. Inj dexamethasone 0.1 mg /kg was also given.

The standard marking for the Alkayat-Bramley incision given over the right side deepened, followed by exposure of bony ankylotic TMJ mass on the right side. Subsequently, the TMJ mass was released with the piezoelectric unit. A right-side coronoidectomy was done as well as a left side after mandibular vestibular incision from 74 to the distal of the ramal region of the left side. Mouth opening of 35 mm was achieved. The CCG was harvested from the seventh rib on the right side. Inter-positional gap arthroplasty was done over the right side with a CCG and temporalis muscle graft. The CCG adapted to the mandibular ramal region of right side using two titanium screws. The temporalis muscle graft was placed between the glenoid fossa and the CCG. Minivac drains were left in situ over the right temporal region. Closure is done in layers. Hemostasis was achieved, and pressure dressing was given over the right material and costochondral site regions.

After surgical release, the patient had a mouth opening of two fingers. Extubation was planned once the patient was fully awake and taking spontaneous breaths. Before extubation, both nasal and oral suction were done. A tongue tie was done before extubation as the patient had a snoring history at night. Extubation was done in the left lateral position. Then, the patient was kept in a lateral position for a few minutes, and oxygen saturation and breathing were observed. Then, the patient was shifted to pediatric ICU. Both normal endotracheal tube (ETT) and flexometallic tube of sizes 4, 4.5, and 5 were kept ready, along with a cricothyrotomy trolley, and the ENT surgeons were on standby in case of emergency. Then, the patient was placed in a lateral position to avoid aspiration. Then, the patient was handed over to a pediatric ICU resident for post-op monitoring, where patient vitals were monitored including SpO_2_, consciousness, and breathing. Post extubation, a tongue tie was done to prevent tongue fall. Physiotherapy began four days following surgery, and thorough follow-up included serial mouth-opening checkups.

## Discussion

TMJ ankylosis is a substantial disabling condition that has a massive effect on the clinical condition, with declining effects on oral hygiene, speech, growth, and in severe cases, micrognathia, which can lead to sleep apnea. TMJ ankylosis of one side of the face frequently leads to facial disproportion due to displacement of the chin towards the afflicted side, which commonly has restricted mandibular growth. The condition can be defined based on the location, the kind of tissue involved (fibrous or bony), or the extent of fusion. TMJ ankylosis was further categorized as real ankylosis, when a condition like infection or trauma promotes bony or fibrous adhesions inside the TMJ capsule, where joint movement is constrained by diseases unrelated to the joint components, such as neurological and muscular disorders [8-10].

In a study of 31 patients aged 3-45 years, with ankylosis of TMJ, Kulkarni et al. found that blind nasal intubation under deep plane anesthetic was effective in 87% of cases, guided by breath sounds and moderate neck motions [11]. Shah et al. treated ankylosis of TMJ as well as sleep apnea (obstructive) by superior laryngeal nerve block, nasal tracheal intubation (blind awake), and local anesthetic solution [12]. The blind approach always increases the risk of damaging the oral mucosa and causing bleeding. Mohan et al. employed complete IV anesthesia with propofol infusion at 50 μg/kg/min and maintained the patient spontaneously using nasal prongs. An extraoral approach was used, and condylectomy was done following local infiltration of the region using a local anesthetic solution [13]. However, FO nasal intubation is a gold standard treatment, despite being fraught with issues.

Gupta et al. employed dexmedetomidine (DXM) to provide sedation during FO nasotracheal intubation (FNI) in ankylosis patients. The DXM group was revealed to have improved intubating conditions and fewer side effects compared to the propofol group. U.S. FDA approved DXM for sedation in people undergoing mechanical breathing for less than 24 hours. Nevertheless, it is utilized as an off-label supplementary medication for analgesia and sedation in pediatric patients, as well as sedation during radiological examination in non-invasive techniques [14].

In another case, the authors utilized adult FO and ETT through each nostril and intubated the trachea with the use of a video camera because pediatric FOB was not available. This approach was employed in two instances. The first case involves TMJ with a 5 mm mouth opening with glossoptosis, while the second case presents ankylosis (post-traumatic) with no mouth opening at three years old. Since performing awake FO intubation is not practical in children, an alternative is to provide an inhalational medication while preserving spontaneous respiration. However, once the mask is removed, sevoflurane ventilation ceases, leaving only a limited amount of time to secure the airway. Therefore, a switch to halothane or isoflurane following induction was done. ETT of smaller size might be used to help maintain airway patency, give oxygen as a nasopharyngeal airway, and function as a conduit for supplying anesthetic gases to maintain appropriate depth during the treatment [15].

Similarly, Fiadjoe et al. performed a comparable approach to protect and secure the airway in a five-year-old child with bilateral TMJ, moderate retrognathia, and a 5 mm mouth opening. Blind nasal intubation was not an option for a child of 2.5 years with TMJ ankylosis on the right side and moderate retrognathia due to swollen adenoids. After inducing anesthesia with halothane, an adult FO was administered orally, and the glottis was seen. An uncuffed ETT of 4.5 mm was then delivered into the trachea orally [16].

## Conclusions

Difficult airway management in pediatrics is very difficult, requiring expertise and proper preparation to cope with any problems that may arise as a result of attempted intubation, such as hemorrhage, trauma, laryngospasm, and hypoxemia. This is a case of TMJ in a pediatric patient and was appropriately managed and treated by a professional team.
